# An Automated Method for the Determination of Intestinal Disaccharidase and Glucoamylase Activities

**DOI:** 10.1155/JAMMC/2006/93947

**Published:** 2006

**Authors:** Zhaoping He, Laura Bolling, Dalal Tonb, Tracey Nadal, Devendra I. Mehta

**Affiliations:** 1 Nemours Biomedical Research Alfred I. duPont Hospital for Children Wilmington DE 19803 USA; 2 Nemours Clinical Management Program Orlando FL 32801 USA

## Abstract

Determination of disaccharidase and glucoamylase activities is important for the diagnosis of intestinal diseases. We adapted a widely accepted manual method to an automated system that uses the same reagents reaction volumes, incubation times, and biopsy size as the manual method. A dye was added to the homogenates as the internal quality control to monitor the pipetting precision of the automated system. When the automated system was tested using human intestinal homogenates, the activities of all the routinely tested disaccharidases, including lactase, maltase, sucrase, and palatinase, as well as the activity of glucoamylase, showed perfect agreement with the manual method and were highly reproducible. The automated analyzer can perform the same routine assays of disaccharidases and glucoamylase with high consistency and accuracy and reduce testing costs by performing a larger sample size with the same number of staff. Additional developments, such as barcoding and built-in plate reading, would result in a completely automated system.


Hindawi Publishing Corporation
Journal of Automated Methods and Management in Chemistry
Volume 2006, Article ID 93947, Pages 1–4
DOI 10.1155/JAMMC/2006/93947

### An Automated Method for the Determination of Intestinal Disaccharidase and Glucoamylase Activities

**Zhaoping He,[1] Laura Bolling,[1] Dalal Tonb,[1] Tracey Nadal,[1] and Devendra I. Mehta[2]**

[1] *Nemours Biomedical Research, Alfred I. duPont Hospital for Children, Wilmington, DE 19803, USA*
[2] *Nemours Clinical Management Program, Orlando, FL 32801, USA*

Received 23 January 2006; Revised 3 March 2006; Accepted 10 April 2006

Determination of disaccharidase and glucoamylase activities is important for the diagnosis of intestinal diseases. We adapted a widely accepted manual method to an automated system that uses the same reagents reaction volumes, incubation times, and biopsy size as the manual method. A dye was added to the homogenates as the internal quality control to monitor the pipetting precision of the automated system. When the automated system was tested using human intestinal homogenates, the activities of all the routinely tested disaccharidases, including lactase, maltase, sucrase, and palatinase, as well as the activity of glucoamylase, showed perfect agreement with the manual method and were highly reproducible. The automated analyzer can perform the same routine assays of disaccharidases and glucoamylase with high consistency and accuracy and reduce testing costs by performing a larger sample size with the same number of staff. Additional developments, such as barcoding and built-in plate reading, would result in a completely automated system.

Copyright © 2006 Zhaoping He et al. This is an open access article distributed under the Creative Commons Attribution License, which permits unrestricted use, distribution, and reproduction in any medium, provided the original work is properly cited.

### 1. INTRODUCTION

Disaccharidases and glucoamylase are localized in the luminal brush-border membrane of intestinal epithelial cells. They are responsible for the digestion of disaccharides. Higher enzyme activity does not have any known clinical significance. However, decreased enzyme activity results in a digestive defect of disaccharide, which clinically may result in osmotic diarrhoea, crampy abdominal pain, or gaseousness. The decreased enzyme activity can be the consequence of generalized intestinal damage or can be an isolated (congenital or acquired) enzyme defect. Measurement of disaccharidases and glucoamylase is helpful in the diagnosis and treatment of digestive and intestinal diseases. Dahlqvist initially developed a manual method to assay disaccharidase activity in homogenates of human intestinal biopsies [1, 2]. This method, although labor-intensive and time-consuming, is currently recognized as the reference for routine disaccharidase assay and is used by most clinical and diagnostic laboratories. The goal of this study was to develop an automatic analyzer for five routinely assayed enzymes: lactase, maltase, sucrase, palatinase, and glucoamylase.

### 2. MATERIALS AND METHODS

### 2.1. *Materials and reagents*

Remnant homogenates from duodenal biopsies of patients were pooled together with no individual identification associated. The homogenates were split equally into two parts, and the assays were run manually and automatically. Lactose, maltose, palatinose, and glycogen (type II from oyster) were from Sigma (St. Louis, Mo). Sucrose was from Mallinkrodt (Paris, Ky). The glucose oxidase reagent was from Pointe Scientific, Inc. (Canton, Mich).

### 2.2. *Manual method*

The manual method was modified from Dahlqvist's [1, 2] to a micromethod suitable for using a microplate reader for final reading. Briefly, 100 $\mu$L of substrate at 56 mM was added to 40 $\mu$L of homogenate (20 $\mu$L for sucrase and maltase) and incubated 60 minutes at 37°C (15 minutes for maltase). The reactions were terminated by incubating at 100°C for 5 minutes. The liberated glucose was measured by

Table 1: Interassay precision of disaccharidase analyzer.

| Enzymes | No. of assays | Mean ($\mu$mol/min/g) | Range ($\mu$mol/min/g) | *CV (%) (SD/Mean) |
|---|---|---|---|---|
| Lactase | 5 | 15.705 | 14.8–17.1 | 7% |
| Maltase | 5 | 172.30 | 163.4–186.7 | 6% |
| Sucrase | 5 | 54.24 | 48.7–61.2 | 9% |
| Palatinase | 5 | 10.81 | 8.9–12.7 | 13% |
| Glucoamylase | 5 | 16.41 | 14.9–17.5 | 6% |

*CV = coefficient of variation, SD = standard derivation.

reaction with 1.0 mL glucose oxidase reagent. After 20 minutes at room temperature, 250 $\mu$L of each final reaction mixture was transferred to a microplate and read at 505 nm with a reference reading at 655 nm. The liberated glucose concentration was then determined using a glucose standard. The final enzyme activity was calculated by subtracting a blank sample in which the homogenates were incubated at 100°C to inactivate the enzymes before addition of substrate. Duplicates were performed for each sample, including two assays and two blanks and standards.

### 2.3. Instruments and robotic method

Our robotic liquid handling system was custom-designed and built by Sias (Eichtal CH-8634, Hombrechtikon, Switzerland). It has a temperature-controlled reagent trough and rack incubation blocks at 4°C, 37°C, and 100°C, as well as room temperature stations. Its robotic arm manipulates racks and has four individually mobile pipette tips. A personal computer that runs the WinRufas software program (Qiagen Instruments AG, Hombrechtikon, Switzerland) controls the robot. The reactions were performed in a 96-tube rack with the same volumes of reagents and homogenate as the manual method.

Remnant homogenate samples (maximum, six samples per run) and four serially diluted glucose standards were stationed in the 4°C blocks, the five sugar substrates were placed in the 4°C troughs, and the glucose oxidase reagent was in the room temperature trough. As a first step, the robot pipetted the homogenates and substrates into the reaction tubes, all at 4°C. Next, the robot moved the 96-tube rack to the 37°C block to start the timed enzymatic reactions (15 minutes for maltase or 60 minutes for the others). The reaction was stopped by moving the rack to the 100°C block for 5 minutes, then after cooling to room temperature, 1.0 mL of glucose oxidase reagent was added. Finally, after mixing, the robot transferred 250 $\mu$L of the final reaction mixture to a 96-well microplate. On completion, the absorbance readings for both the manual and automatic assays were performed on a BioRad Model 550 microplate reader. Assays and blanks for each enzyme reaction were performed in duplicates.

### 2.4. Internal control for the robotic method

An internal control marker, p-nitrophenol (pNP), was added to the homogenate to quantify the accuracy and consistency of the robotic pipette system. P-nitrophenol has a peak at 405 nm, thus, it does not interfere with the glucose oxidase reagent reaction reading at 505/655 nm. In addition, pNP does not alter the enzymatic activities of the five enzymes. At the end of the reaction, the plate was read at 505/655 nm and 405 nm. The variation within the duplicate sample readings at 505/655 nm and 405 nm was calculated to determine accuracy and consistency.

### 2.5. Data analysis

All data were on the interval level of measurement (lactase, maltase, sucrase, palatinase, glucoamylase). Therefore, interrater agreement (manual versus robot methods) was assessed using intra-class correlation coefficients (ICCs) [3]. The ICCs were computed for consistency using a two-way random effects model as specified by McGraw and Wong [4].

### 3. RESULTS

### 3.1. Precision of automated method

Enzymatic activities of lactase, maltase, sucrase, palatinase, and glucoamylase were measured in five different runs on the same pool of human intestinal homogenates. Results in Table 1 show the mean activities and the range of the measurements. Reproducibility was assessed by the coefficient of variation. The results indicated the automated method was highly reproducible for human intestinal biopsies.

### 3.2. Correlation with the manual method

The measurements of the four disaccharidases (lactase = L, sucrase = S, maltase = M, palatinase = P) and glucoamylase (G) with the robotic method were compared with the manual method in 66 paired runs. For each paired run, the same pool of homogenates was equally split into two parts: one run manually and the other using the automated method. The values obtained by manual assay for the four disaccharidases and glucoamylase were plotted against those of the automated assay (Figure 1). Most of the values, from the very low activities to the high ones, fell linearly (Figure 1). The average measurement ratios of robotic versus the manual method are shown in Table 2. Analysis using intraclass correlation coefficients showed consistency values of 0.96, 0.97, 0.95, 0.99, and 0.98 for lactase, maltase, sucrase, palatinase,

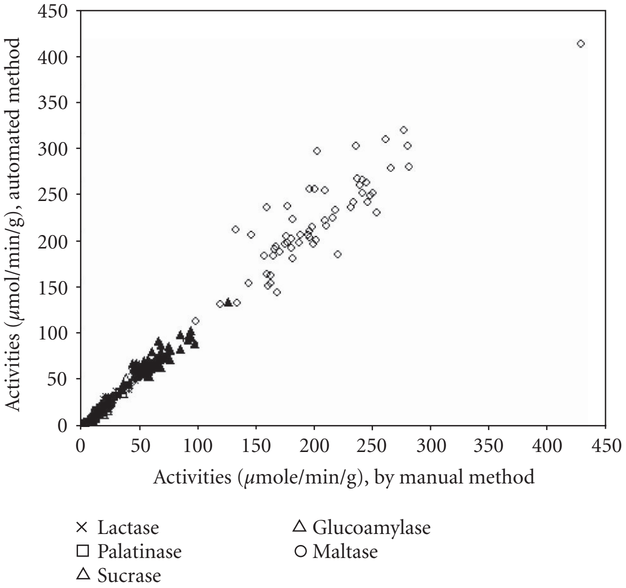

Figure 1: Comparing the activities of lactase, sucrase, maltase, palatinase, and glucoamylase from 66 paired assays of the manual method ($x$-axis) and the automated method ($y$-axis).

Table 2: Comparison of measurements: automatic versus manual. L = lactase, M = maltase, S = sucrase, P = palatinase, and G = glucoamylase.

| | L | M | S | P | G |
|---|---|---|---|---|---|
| Average ratio (automatic/manual) | 1.2 ± 0.2 | 1.1 ± 0.1 | 1.2 ± 0.3 | 1.0 ± 0.2 | 1.1 ± 0.2 |
| Consistency value | 0.96 | 0.97 | 0.95 | 0.99 | 0.98 |

and glucoamylase, respectively. These results showed almost perfect agreement between the two methods (Table 2).

### 3.3. Cost and time savings of the robotic method

The robotic method requires 3 hours to run six patient samples, five enzymes per sample. This method can potentially perform 12 patient samples per day. One person can perform an average of four samples per day manually, including time for calculation and reporting the results. This constitutes substantial savings in time and staffing costs with a three-fold reduction of labor using the automated method when compared with the manual method. This saving will result in a reduction in the overall patient care cost.

### 3.4. Biopsy size limitation

For manual assays, homogenates from biopsies weighing at least 4 mg were brought to a volume of 800 mL. For the automated assays, homogenates from biopsies weighing at least 4 mg were brought to a volume of 880 mL. The robotic method required some dead volume in the tube of homogenate, and this extra volume also served as a backup if the instrument malfunctioned. Therefore, the robotic method does not analyze biopsy samples of less than 4 mg.

### 4. DISCUSSION

The most widely used method to measure disaccharidases and glucoamylase to diagnose digestive diseases was described by Dahlqvist and is based on the measurement of liberated glucose with a glucose oxidase reagent [1, 2]. This assay requires specifically trained staff and is typically beyond the means of a clinical laboratory, as it requires resources of a specialty laboratory. After first adapting Dahlqvist's protocol to a manual micromethod, the method was then adapted to an automated analyzer, which can measure a panel of five enzymes for up to 12 patient samples in one day. This method showed high reproducibility when tested with human intestinal samples. In addition, the measurements of the enzymes demonstrated near perfect agreement with the manual method that is now widely used in most clinical laboratories. Sall and Ferard [5] reported results of a continuous, automated method using glucose dehydrogenase to detect liberated glucose. However, their method cannot accurately measure lactase, one of the most important disaccharidases for diagnosis of intestinal disease. In addition, no patient samples were tested with their automated method. The change in reporting required for Sall-Ferard continuous assay also required clinicians to use unfamiliar values and norms. The automated method developed by the authors uses the same reagent and sample volumes as the method routinely performed in most clinical laboratories; therefore, any laboratories currently using Dahlqvist's manual method [1, 2] can change to the automated method without establishing new cutoff values for diagnosis or testing new reagents. After the initial purchasing investment, the automated assay will reduce testing costs because a larger sample size can be performed per person in one day. As for the biopsy size limitation, most laboratories require a biopsy size of 4.0 mg to measure all five enzymes using the manual method. Therefore, this limitation will not be an obstacle when changing to the automated assay. Finally, barcoding samples and adding the plate-reader step into the automated routine would further simplify the process and yield a stable, serviceable analyzer that could be used in any clinical laboratory.

### ACKNOWLEDGMENT

This study was conducted at the Alfred I. duPont Hospital for Children in Wilmington, Delaware, USA.

### REFERENCES

[1] A. Dahlqvist, "Assay of intestinal disaccharidases," *Analytical Biochemistry*, vol. 22, no. 1, pp. 99–107, 1968.

[2] A. Dahlqvist, "Assay of intestinal disaccharidases," *Scandinavian Journal of Clinical and Laboratory Investigation*, vol. 44, no. 2, pp. 169–174, 1984.

[3] J. R. Landis and G. G. Koch, "The measurement of observer agreement for categorical data," *Biometrics*, vol. 33, no. 1, pp. 159–174, 1977.

[4] K. O. McGraw and S. P. Wong, "Forming inferences about some intraclass correlation coefficients," *Psychological Methods*, vol. 1, no. 1, pp. 30–46, 1996.

[5] I. Sall and G. Ferard, "A continuous automated method for the determination of intestinal disaccharidase activities," *Digestion*, vol. 59, no. 6, pp. 703–707, 1998.

